# Alzheimer's disease: new diagnostic and therapeutic tools

**DOI:** 10.1186/1742-4933-5-7

**Published:** 2008-08-13

**Authors:** Marco Racchi, Daniela Uberti, Stefano Govoni, Maurizio Memo, Cristina Lanni, Sonya Vasto, Giuseppina Candore, Calogero Caruso, Loriana Romeo, Giovanni Scapagnini

**Affiliations:** 1 Department of Experimental and Applied Pharmacology, University of Pavia, Italy; 2 Department of Biomedical Sciences and Biotechnologies, University of Brescia, Italy; 3 Immunosenescence Unit, Department of Pathobiology and Biomedical Methodologies, University of Palermo, Italy; 4 Neurological Science Institute, National Research Council, Catania, Italy; 5 Department of Health Sciences, University of Molise, Campobasso, Italy

## Abstract

On March 19, 2008 a Symposium on Pathophysiology of Ageing and Age-Related diseases was held in Palermo, Italy. Here, the lectures of M. Racchi on History and future perspectives of Alzheimer Biomarkers and of G. Scapagnini on Cellular Stress Response and Brain Ageing are summarized. Alzheimer's disease (AD) is a heterogeneous and progressive neurodegenerative disease, which in Western society mainly accounts for clinica dementia. AD prevention is an important goal of ongoing research. Two objectives must be accomplished to make prevention feasible: i) individuals at high risk of AD need to be identified before the earliest symptoms become evident, by which time extensive neurodegeneration has already occurred and intervention to prevent the disease is likely to be less successful and ii) safe and effective interventions need to be developed that lead to a decrease in expression of this pathology. On the whole, data here reviewed strongly suggest that the measurement of conformationally altered p53 in blood cells has a high ability to discriminate AD cases from normal ageing, Parkinson's disease and other dementias. On the other hand, available data on the involvement of curcumin in restoring cellular homeostasis and rebalancing redox equilibrium, suggest that curcumin might be a useful adjunct in the treatment of neurodegenerative illnesses characterized by inflammation, such as AD.

## Background

On March 19, 2008 a Symposium on Pathophysiology of Ageing and Age-Related diseases was held in Palermo, Italy. Here, the lectures of M. Racchi on History and future perspectives of Alzheimer Biomarkers and of G. Scapagnini on Cellular Stress Response and Brain Ageing are summarized.

Alzheimer's disease (AD) is a progressive neurodegenerative disorder affecting aged people; AD prevalence is approximately 1% between 65 and 69 years and is higher than 50% in individuals above 95 years. It is characterized by irreversible cognitive and physical deterioration. With increasing life expectancy across the world, dementia is a rapidly growing socioeconomic and medical problem. The confirmatory diagnosis of AD is based on the recognition and quantification of senile plaques and neurofibrillary tangles, which are the hallmarks of the disease [1].

AD prevention is an important goal of ongoing research. Two objectives must be accomplished to make prevention feasible: i) individuals at high risk of AD need to be identified before the earliest symptoms become evident, by which time extensive neurodegeneration has already occurred and intervention to prevent the disease is likely to be less successful and ii) safe and effective interventions need to be developed that either reduce or slow the accumulation of AD neuropathology or lead to a decrease in clinical expression of this pathology [2].

### p53 as a putative peripheral marker for AD

The treatment of AD remains a major challenge because of an incomplete understanding of the events that lead to the selective neurodegeneration typical of Alzheimer's brains. Nowadays the attention is focused on one side on the study of the β-amyloid (Aβ) precursor protein (APP) metabolism's pharmacological modulation, and on the other one to develop disease-modifying or -arresting compounds. The first purpose is that to reduce the development of Aβ in the hope of reducing the formation of a potentially neurotoxic peptide whereas examples of the second concern the use either of monoclonal antibodies direct to inflammatory mediators or of β-sheet breakers [2-6].

In view of existing and emerging therapeutic compounds, the focus has increasingly shifted to accurate detection of the earliest phase of illness and, to date, there is an increasing interest to develop techniques allowing an accurate detection of the earliest stages of the disease. Candidate biochemical markers for AD should be molecules representing some of the cerebral pathogenetic processes typical of AD or representing altered metabolic or cellular processes as shown by several studies performed either on brain or on peripheral tissues from affected patients. A wide variety of different proteins such as inflammatory markers, markers of oxidative stress, apolipoproteins, and markers of neuronal degeneration in blood and cerebrospinal fluid (CSF) have been examined [7,8]. Most of these studies have, however, yielded conflicting results. The cerebrospinal fluid has been the focus of research for diagnostic markers in AD pathology due to its direct contact with the extracellular space of the brain [7]. The more encouraging results come from the studies on the measurement of different isoforms of Aβ in CSF, particularly Aβ 1–42 [9], due to its role in the early pathogenesis of AD. Most studies showed that Aβ 1–42 concentrations are lower in the CSF of AD [7,8]. Unfortunately, plasma Aβ 1–40 and Aβ 1–42 did not correlate with the disease. In fact the results from these studies are often contradictory [10].

The biological markers can be classified as primary (specific), such as Aβ, or secondary to the disease, or they can simply be epiphenomenal in nature. In search of secondary markers, Uberti et al. demonstrated an intriguing correlation between p53 and AD by using cell lines derived from these patients [11]. Fibroblasts of sporadic AD patients represent an important starting point in the research for novel biomarkers because of their various abnormalities in metabolic and biochemical processes, which reflect some of the events in the AD brain. They described and demonstrated an abnormal response of AD fibroblasts to an acute oxidative injury; in particular, fibroblasts from AD patients were found to be less vulnerable to the oxidative injury induced by H_2_O_2 _in comparison with fibroblasts from non-AD subjects. On the basis of immunoprecipitation studies with conformation-specific p53 antibodies, which discriminated folded vs. unfolded p53 tertiary structure, they found that in fibroblasts from AD patients a significant amount of total p53 assumes an unfolded tertiary structure in comparison with fibroblasts from control elderly subjects. Sequence analysis of the p53 gene allowed to exclude the possibility that the mutant p53 found in AD fibroblasts was the result of gene mutation. Thus, these data suggest that one of the peripheral events associated to the disease is responsible for generating such p53 isoform [11,12].

In the attempt of investigating on the mechanism of such alteration, they assessed the contribution of APP metabolic products to the change in p53 conformational state. They found that the exposure to nanomolar concentrations of beta-amyloid (Aβ) 1–40 peptide induced the expression of an unfolded p53 protein isoform in fibroblasts derived from non-AD subjects. These data suggest that the tertiary structure of p53 and the sensitivity to p53-dependent apoptosis are influenced by low concentrations of soluble Aβ. On this basis, they hypothesised that low amounts of soluble Aβ induce early pathological changes at cellular level that may precede the amyloidogenic cascade. One of these changes is the induction of a novel conformational state of p53 [11,13].

In addition and most importantly, Lanni et al. [14] were able to develop a rapid, easy and quantitative flow cytometric approach for the discrimination of conformational mutant p53-bearing cells from AD patients compared to non-AD controls, using small volumes of blood. Using this technique, they processed 75 AD, 66 controls, 15 subjects affected by another neuroinflammatory disease, Parkinson's disease and 3 subjects affected with other types of dementia (2 vascular dementia; 1 progressive supranuclear palsy) and confirmed the previous findings: AD subjects expressed higher levels of unfolded p53 in comparison with controls and subjects with other neurological diseases. The levels of conformationally altered p53, both in controls and AD patients, correlated with age but not with the lenght of illness or with the Mini Mental State Examination value. Interestingly, the sensitivity and specificity within different age intervals were more significant in subjects up to 70 years of age compared with the corresponding values for individuals older than 70 years. Within this specific age interval (≤ 70 years), the Authors worked out a sensitivity of 90% to discriminate AD patients from nondemented aged individuals at a specificity value of 77%. A comparison of these sensitivity and specificity values with those published in several studies, which evaluated the diagnostic power of CSF markers for AD (Total-tau, Phospho-tau and Abeta 1–42), reveal that p53 measurement is more sensitive (90% compared to respectively 81.4%, 81.3% and 85.9%), but less specific (77% compared to respectively 91.5%, 91.2% and 88.5%) [7].

On the whole, these data strongly suggest that the measurement of conformationally altered p53 in blood cells has a high ability to discriminate AD cases from normal ageing, Parkinson's disease and other dementias. In spite of the fact that the method described in this study has a lower specificity value compared to CSF biomarkers, its high sensitivity in subjects up to 70 years and the non invasive nature of the test, permit its proposal as an adjunctive marker. Accordingly, p53 analysis may be used in the clinical evaluation of mild cognitive impairment cases or to improve a clinical diagnosis of AD, which should be based on cumulative information derived from clinical examination, brain neuroimageing techniques and biochemical markers either from CSF or blood. In a disease where therapeutic treatments are at most symptomatic, early treatment and therefore early prediction of future pathology is particularly important. Whether this different expression of conformationally altered p53 will be suitable as an adjunctive diagnostic tool in early stage AD in larger and independent populations of patients is matter of further investigations.

On the other hand, p53 is a hot topic in AD research. Interestingly, it has been hypothesised that oxidative modification of p53 could be involved in the neuronal loss observed in neurodegenerative conditions [15,16].

### Cellular stress response

Oxidative stress has been implicated in a variety of pathophysiological conditions, including neurodegenerative disorders. Irrespective of the source and mechanisms that lead to the generation of reactive oxygen species, mammalian cells have developed highly regulated inducible defensive systems, whose cytoprotective functions are essential in terms of cell survival. When appropriately activated, each one of these systems has the possibility to restore cellular homeostasis and rebalance redox equilibrium. Activation of antioxidant pathways is particularly important for tissue with relatively weak endogenous antioxidant defenses, such as the brain. Increasing evidences, in fact, support the notion that reduction of cellular expression and activity of antioxidant proteins and consequent augment of oxidative stress are fundamental causes for aging processes and neurodegenerative diseases [17]. Among the molecules belonging to stress protein family, Heme oxygenase-1 (HO-1) has been the object of intensive studies in the brain for its potential role in protecting neurons against cell death. HO enzymes provide the first and rate-limiting step in heme degradation, to give biliverdin, gaseous carbon monoxide and free iron. All the byproducts of HO activity play a significant role in physiological cell functions [18]. In the CNS, the HO system has been reported to be very active [19,20] and its modulation seems to play a crucial role in the pathogenesis of neurodegenerative disorders. Deregulation of the HO system has been associated with the pathogenesis of Alzheimer's disease, multiple sclerosis and brain aging [21,22]. Many studies clearly demonstrate that activation of HO-1 in neurons is strongly protective against oxidative damage and cell death [23]. Thus, modulation of HO-1 should represent a potential pharmaceutical strategy for the treatment of neurodegenerative disorders. A number of experimental and epidemiological studies have recently supported the beneficial effects of some commonly used natural products in preventing various pathologic conditions ranging from cardiovascular diseases to cancer. Spices and herbs often contain phenolic substances with potent antioxidative and chemopreventive properties [24]. Scapagnini et al. have previously shown that curcumin (1,7-bis [4-Hydroxy-3-methoxyphenyl]-1,6-heptadiene-3,5-dione), a natural phenolic agent, extracted from the rhizome of *Curcuma Longa*, strongly induced HO-1 expression and activity in rat astrocytes [25]. The Authors have then extended their findings demonstrating curcumin ability to induce HO-1 in cultured hippocampal neurons [26]. The results indicate that curcumin activates HO-1 and phase II enzymes expression in astrocytes and neurons, probably by activation of transcription factor Nrf2, and this activation is able to effort a significant cytoprotection in cultured neurons exposed to oxidative stress. The involvement of curcumin in restoring cellular homeostasis and rebalancing redox equilibrium, suggests that it might be a useful adjunct also in the treatment of neurodegenerative illnesses characterized by inflammation, such as AD. This idea has been reinforced by epidemiological studies showing that, in India where this spice is widely used in daily diet, there is a reduced age-adjusted prevalence of AD (in patients between 70 and 79 years of age is 4.4-fold less than that of the United States) [27]. Consistent with its possible use in neurodegenerative diseases, curcumin has been reported to decrease oxidative damage and amyloid deposition in a transgenic mouse model of Alzheimer's disease, and to reverse Aβ-induced cognitive deficits and neuropathology in rats [28,29]. Other plant-derived phenolic agents with analogous chemical structures to curcumin have been demonstrated to strongly activate HO-1 expression and to defend cells against oxidative stress. In particular, Scapagnini et al. have shown that ethyl ferulate, resveratrol (a phitoalexin derived from grape) and caffeic acid phenethyl ester (CAPE), are able to protect neurons via HO-1 induction [30]. These and other studies identify a novel class of natural substances that could be used for therapeutic purposes as potent inducers of HO-1 in the protection of tissues against inflammatory and neurodegenerative conditions. It needs to be emphasized that curcumin, and other plant constituents eventually become part of the human diet and can be consumed daily as herbal supplements. Further in vitro and in vivo studies using curcumin-like molecules will give important information on the feasibility of developing new pharmacological strategies for maximizing heme oxygenase activity in targeted tissues as an alternative to or in combination with HO-1 gene therapy.

However, curcumin studies are a growing area in AD research [31] as well as in other pathological conditions. Various preclinical cell culture and animal studies suggest that curcumin has potential as an antiproliferative, anti-invasive, and antiangiogenic agent; as a mediator of chemoresistance and radioresistance; as a chemopreventive agent; and as a therapeutic agent in wound healing, diabetes, AD, Parkinson disease, cardiovascular disease, pulmonary disease, and arthritis [32].

## Conclusion

A major goal of ongoing research in AD is to improve early detection by developing tools to move diagnosis backward in disease temporal course, i.e. before the clinical manifestation of the disease, where a treatment might play a decisive role in preventing or significantly retarding the manifestation of the disease [2,33]. On the whole, data here reviewed strongly suggest that the measurement of conformationally altered p53 in blood cells has a high ability to discriminate AD cases from normal ageing, Parkinson's disease and other dementias. On the other hand, available data on the involvement of curcumin in restoring cellular homeostasis and rebalancing redox equilibrium, suggest that curcumin might be an useful adjunct in the treatment of neurodegenerative illnesses characterized by inflammation, such as AD.

## Competing interests

The authors declare that they have no competing interests.

## Authors' contributions

MR, DU, SG, MM, CL carried out all the studied on conformational p53, SV, GC, CC took care of pharmacogenomic approach and drafted the manuscript, LR, GS carried out the curcumin experiments. All authors read and approved the final manuscript.
